# Lifestyle intervention in early pregnancy can prevent gestational diabetes in high-risk pregnant women in the UAE: a randomized controlled trial

**DOI:** 10.1186/s12884-022-04972-w

**Published:** 2022-08-30

**Authors:** Amena Sadiya, Vidya Jakapure, Ghida Shaar, Rama Adnan, Yohannes Tesfa

**Affiliations:** 1 Rashid Centre for Diabetes and Research, Sheikh Khalifa Medical City, Ajman, PO Box-5166, UAE; 2 Education, Training and Research Department, Sheikh Khalifa Medical City, Ajman, UAE

**Keywords:** Dietary, Type 2 diabetes, Gestational diabetes mellitus, United Arab Emirates

## Abstract

**Purpose:**

A prevalence of gestational diabetes mellitus (GDM) is approximately three times higher than the global rate in the UAE. However, it has not yet been studied whether a 12-week moderate-intensity lifestyle intervention can prevent gestational diabetes among pregnant women at high risk in this region.

**Patients and methods:**

A pragmatic, open-label, randomized clinical trial was conducted.

Sixty-three women aged 18 to 45 years, with ≤12 weeks of gestation, singleton pregnancy, and having ≥ two risk factors for GDM were randomly assigned to the Lifestyle Intervention (LI) group (*n* = 30) or Usual Care (UC) group (*n* = 33). The women in the LI group received a 12-week, moderate-intensity lifestyle intervention with individualized counseling on a diet, physical activity, and behavior change by a licensed dietitian. The women in the UC group received usual antenatal care. The primary outcome was the incidence of GDM based on the IADPSG criteria at 24-28 weeks of gestation.

**Results:**

The incidence of GDM was 33.3% in LI group and 57.5% in UC group. The crude relative risk (RR) for GDM was 0.59 (95% CI, 0.32-1.04, *p* = 0.05). The multivariable logistic regression model without adjustment showed OR = 0.37 (95% CI, 0.13-1.02, *p* = 0.05) and after adjusting with Age, BMI and family history of diabetes reported OR = 0.26, 95%CI 0.07, 0.92, *p* = 0.04. in LI vs UC. The daily dietary intake of calories (− 120 kcal, *p* = < 0.01), carbohydrates (− 19 g, *p* < 0.01), and fat (− 5 g, *p* = 0.03) was reduced, and physical activity time (+ 52 min, *p* = 0.05) increased in the LI group after the intervention. However, the LI had no significant effect on maternal and neonatal outcomes.

**Conclusion:**

A 12-week moderate intensity lifestyle intervention in early pregnancy could reduce the relative risk of GDM by 41% among high-risk pregnant women in the UAE. These findings could impact public health outcomes in the region.

**Trial registration:**

Trial registration Retrospectively registered NCT04273412*,18/02/2020*.

## Introduction

Gestational diabetes mellitus (GDM) is a common pregnancy complication in which spontaneous hyperglycemia develops, posing a severe threat to maternal and child health. As per the International Association of Diabetes and Pregnancy Study Groups (IADPSG) criteria, globally 14.7%, while in the United Arab Emirates (UAE), 45.3% of pregnancies are diagnosed with GDM [1–3].

The effects of GDM can be short- and long-term for both the mother and the child. Specifically, high rates of pre-eclampsia, pre-term labor, operative deliveries, macrosomia, fetal hypoglycemia, perinatal jaundice, birth injury, and a greater risk of developing type 2 diabetes mellitus (T2D) [4]. It has been estimated that, according to the International Diabetes Federation (IDF), half of women who have had GDM could develop T2D within five to ten years after delivery. Moreover, exposure to hyperglycemia in the womb predisposes the fetus to an increased risk of being overweight or obesity, which is associated with risk of T2D [5].

Gestational diabetes mellitus is a heterogeneous disorder stemming from a complex interaction between genetic, physiological, and environmental risk factors [6]. As pregnancy progresses, a surge of local and placental hormones promotes a state of insulin resistance, leading to glucose intolerance [4]. The environmental factors include obesity, excessive gestational weight gain, advanced maternal age, family history of T2D, history of GDM, polycystic ovarian syndrome, westernized diet, and ethnicity [4, 7, 8].

Lifestyle intervention has been found to be effective in preventing T2D in individuals with impaired glucose tolerance (“high-risk individuals”) [9–11]. Studies have indicated that the success of such interventions can be attributed to a comprehensive approach (dietary modification, increased physical activity, and weight reduction for overweight), which simultaneously addresses lifestyle-related risk factors [11]. Since GDM and T2D are closely related in risk factors, etiology, and pathogenesis, it is anticipated that lifestyle interventions may impact the onset of GDM in high-risk women. Nevertheless, there were inconsistent results across several studies due to the heterogeneity of the population, the type of intervention, the duration of intervention, and gestation age during intervention [12]. Findings from 19 randomized controlled trials studies showed a possible reduction in GDM by 15% in women who received diet and exercise programs compared to standard care. However, similar results were not evident with exclusive dietary advice or physical activity interventions [12].

Due to its high prevalence, GDM has become an increasingly significant public health problem in the UAE**.** Identifying interventions for the prevention of GDM becomes increasingly important. To our knowledge, we have not found any randomized clinical studies that have investigated the effectiveness of lifestyle interventions in preventing GDM in high risk pregnant women in this region. Thus, this pragmatic, randomized, clinical study evaluated whether moderate-intensity lifestyle interventions including dietary change, moderate physical activity, and behavior modification may reduce the incidence of GDM in high risk pregnant women in the UAE.

## Material and methods

### Study design

A randomized open-label, pragmatic, clinical trial with two arms was conducted in Sheikh Khalifa Medical City Ajman, UAE; between October 2018 and August 2020. Participants were recruited from the prenatal clinic at Sheikh Khalifa Hospital-Women and Children, and the intervention group was enrolled at Rashid Centre for Diabetes and Research. A Research Ethics Committee of the Ministry of Health and Prevention, UAE (reference no. MOHP/REC-16/2018) approved this study as per the Declaration of Helsinki while the protocol was retrospectively registered on ClinicalTrials.gov. (NCT04273412, 18/02/2020).

### Study participants

Eligible pregnant women were aged 18 to 45 years, with ≤12 weeks of gestation, singleton pregnancy, and having ≥ two risk factors for GDM (high-risk ethnic group (Middle Eastern, Southern Asian) first-degree relative with T2D, pre-pregnancy Body mass index (BMI) ≥ 30 kg/m^2^, previous macrosomic baby weighing > 4.5 kg, history of GDM or polycystic ovarian syndrome) [13]. The exclusion criteria were, pre-existing diabetes, fasting blood glucose ≥126 mg/dl or Hba1c ≥ 5.8% at first prenatal visit, on medications interfering with glucose metabolism (corticosteroids, metformin), psychiatric disorders, hypertension or medical conditions preventing from any physical exercise, unable to comprehend or cope with program requirements, unable to give informed consent, or with ≥2 consecutive first trimester abortions who conceived spontaneously.

Women who were eligible for the study voluntarily participated, signed an informed consent form, and were allowed to discontinue the study at any time after participating. The randomization process was performed by study staff with computer-generated random numbers, which were randomly assigned in blocks of four and were concealed from recruiters. Participants were allocated to the Lifestyle Intervention (LI) group or the Usual Care (UC) group (1:1). Participants, and dietitians were aware of the allocation assignments, but the statistician and research assistant remained blinded in accordance with the design of the RCT [14].

We estimated a sample of 35 participants in each arm to detect the difference in the incidence of GDM between the intervention (15%) and control (45.3%) groups of 30% (2-tailed, α error of 0.05) gave power of 80%) [3, 14]. We assumed a 10% dropout rate. However, with a sample size of 63 we achieved a power of 75%.

### Outcomes and data collection

A primary outcome of this study was to determine the incidence of GDM in the two arms. It was determined through an oral glucose tolerance test (OGTT) conducted at 24 to 28 gestation weeks. The diagnosis of GDM was defined by the IADPSG criteria, when any one or more of the following cut-offs for blood glucose is met i.e. ≥ 92 mg/dl (≥ 5.2 mmol/l) fasting or 1-hour ≥180 mg/dl (≥ 10 mmol/l) or 2-hour ≥153 mg/dl (≥ 8.5 mmol/l). The secondary outcomes were gestational weight gain (GWG), fetal birth weight and mode of delivery. The total GWG is the weight gain from preconception to 35-37 weeks of gestation. The pre-gestation weight was self-reported and was used to calculate pre-pregnancy BMI; this remains subjective; however, its validity is verified in earlier studies on GWG [15].

Data were collected at baseline (recruitment), prior to intervention, post-intervention, and during delivery. In order to collect information on demographics, anthropometry, medical, obstetric history, supplements, medications, allergies, and use of supplements, questionnaires were utilized. Information on secondary outcomes was derived from antenatal clinic records. A licensed dietitian recorded both groups’ dietary intake and physical activity data. A standardized tool of 24- hour food recall and frequency questionnaire (developed for the study) were used as a means of recording the dietary information [16]. In addition, physical activity was evaluated using self-reported activity or steps recorded using a mobile phone pedometer application in accordance with the evaluation of physical activity.

### Intervention

In the UC group, women received standard antenatal care, including general advice regarding lifestyle changes, and returned for data collection appointments 12 weeks after enrolling. In contrast, women in the LI group received a 12-week moderate-intensity lifestyle intervention beginning on the recruitment day in their first trimester. Compared with other interventions reported in the literature, the LI component of this study was unique since it was adapted from previous interventions reported on the same population for weight management and diabetes management [17, 18]. The LI included two face-to-face individualized dietary consultations and two telephonic counseling sessions by a licensed dietitian. At recruitment, the first face-to-face consultation occurred and the second was 12 weeks later, while telephone follow-ups were conducted between the two face-to-face. We have suggested a target weight gain in the second and third trimesters based upon recommendations from the Institute of Medicine (IOM); normal weight (1 lb./week), overweight (0.6 lb./week), and obese (includes all classes) (0.5 lb./week) [19]. The dietitians were skilled at providing intervention based on American Diabetes Association (ADA) recommendations using standardized teaching material and follow-up tools. The dietary counseling focused on optimizing participants’ consumption of whole grains, vegetables, fruits, portion control, lowering intake of ultra-processed food, and simple sugars. A macronutrient composition of 50-55% carbohydrates, 25-30% fat, and 20% protein was targeted for the diet [20, 21].

The women were encouraged to increase physical activity to 150 minutes of moderate-intensity activity a week or to monitor a minimum of ten thousand steps per day (1 hour 40 minutes daily activity) [22]. In order to achieve behavior change, motivational interviewing, SMART (specific, measurable, attainable, relevant and time-bound) goal setting, self-monitoring (pedometer, food log), and problem-solving skills were employed in the intervention [18].

### Statistical analysis

In this study, categorical variables are represented by frequency distributions and continuous variables by means and standard deviations. The normality of the variables was tested by using the Shapiro-Milk W test and descriptive analysis is performed based on the distribution of the data. Primary analyses were performed on the basis of intention to treat (ITT). Comparison of groups’ characteristics for categorical variables was done by chi-square tests and student t-tests for continuous variables (or Mann-Whitney U tests for non-normally distributed variables). The magnitude of the association between the groups on binary outcome was assessed using relative risk (RR) with 95% confidence interval (CI). Logistic regression analyses were used to estimate the intervention effect in the unadjusted model, as well as controlling for common confounders such as age, BMI, and a history of T2D. All *p* values are two-tailed, and *P* < 0.05 is regarded as the cut off value for statistical significance. The data were analyzed with Stata Statistical Software: Release 13. College Station, TX: StataCorp LP.

## Results

**Fig. 1:**
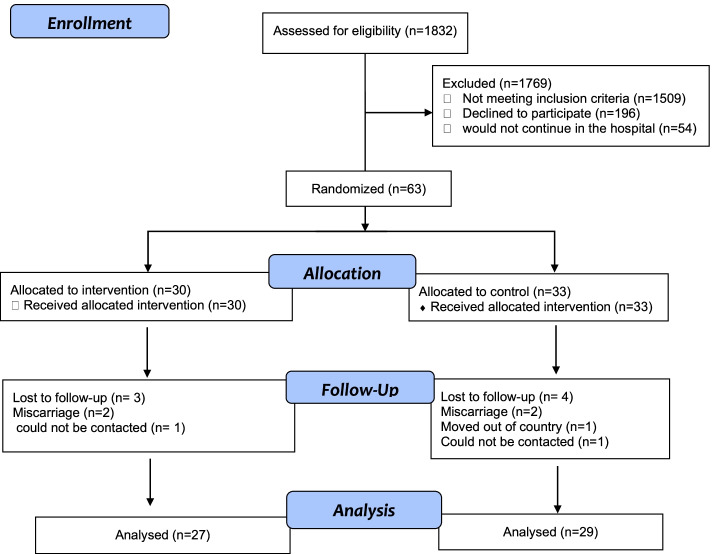
Consort flow chart of the study

In total, 1832 pregnant women were screened, and 323 were eligible (Fig. 1). Among these, 63 (19%) gave informed consent to participate, and were randomly assigned to either the UC group (*n* = 33) or the LI group (*n* = 30). There were very few missing data points since the dropout rate was about 10%.

Both groups were matched by age and BMI; however, all baseline characteristics were comparable between the two groups (Table 1). Two-thirds of the participants in both groups were Middle Easterners while the remaining participants were Asians. It should be noted that the participants in both groups consumed calories, macronutrients, and exercise in relatively similar amounts. None of the participant was smoking or consuming alcohol.

**Table 1:** Baseline demographic and clinical characteristics of the participants in the intervention and control groups

	Lifestyle Intervention group (*n* = 30)	Usual Care group (*n* = 33)	*P* value
Age (years) Mean ± SD	32.8 ± 4.1	30.79 ± 5.2	0.09
Body weight (kg)	72.6 ± 12.5	68.37 ± 14.9	0.23
Height (m)	1.6 ± 0.1	1.57 ± 0.07	0.18
BMI (kg/m2)	28.2 ± 3.9	27.6 ± 6.1	0.65
Gestational age at baseline (weeks)	8.9 ± 2.3	7.8 ± 2.0	0.08
Educational status at baseline, n (%)			0.43
Primary education	3(10)	6(18)	
Higher secondary school	9(30)	10(30)	
Graduation	11(37)	10(30)	
Higher education	7(23)	7(21)	
Previous deliveries, n (%)			0.07
None	4	9	
1	6	12	
2	5	3	
3+	15	9	
Prior GDM, n (%)	21 (70)	17 (51.5)	0.10
Blood pressure Systolic (mmHg)	115 ± 8	113 ± 12	0.66
Blood pressure Diastolic (mmHg)	67 ± 8	67 ± 7	0.96
Family h/o DM, n (%)	24 (80)	28 (85)	0.85

*Notes*: Data are mean ± SD unless otherwise indicated

*Abbreviations*: *GDM* Gestational Diabetes Mellitus, *BMI* Body mass index

We recorded the daily dietary intake and physical activity patterns of participants in both groups at baseline and after 12 weeks of intervention. Participants in the LI group showed a greater improvement in their pattern of nutritional intake than participants in the UC group. Table 2 shows that the daily intake of calories (− 120 kcal, *p* = 0.01), carbohydrate (− 19 g, *p* = 0.01), and fat (− 5 g, *p* = 0.03) reduced significantly in the LI group, which could be attributed to decreased consumption of sweets and sweetened beverages. In comparison, no changes were observed in the UC group. However, the difference in the nutritional intake between the groups was not evident statistically. Participant attendance at dietitian consultations was 96.6% in the first session and 65.0% in the second session, and telephone consultations were achieved by 86.6% of participants in the IL group.

**Table 2:** Change in daily nutritional intake and physical activity at baseline and post-intervention

	Lifestyle Intervention Group			Usual Care Group			
	Baseline	Post-intervention	***P***-value	Baseline	Post-intervention	***P***-value	Change ***P***-value
Calorie (kcal)	**1814 ± 432**	**1694 ± 366**	**0.004**	**1870 ± 401**	**1824 ± 360**	**0.25**	**0.23**
Carbohydrate (gm)	**227 ± 66**	**208 ± 53**	**0.009**	**235 ± 58**	**227 ± 51**	**0.21**	**0.93**
Protein (gm)	**60 ± 17**	**59 ± 16**	**0.30**	**62 ± 16**	**62 ± 15**	**0.421**	**0.99**
Fat (gm)	**74 ± 14**	**69 ± 13**	**0.03**	**76 ± 17**	**74 ± 15**	**0.35**	**0.93**
Physical Activity (min/week)	**88 ± 101**	**140 ± 66**	**0.05**	**87 ± 113**	**126 ± 108**	**0.38**	**0.98**

*Notes*: Data are expressed as mean ± SD, p-value between differences in changes from baseline, p-value by independent t test; change *p*-value is the difference within treatment by 2-tailed paired t test

In the IL group, twelve participants (36.6%) demonstrated a high level of compliance with the recommendations. As we began the intervention in the first trimester, we noticed some participants reported nausea, loss of appetite, and vomiting, which influenced  their food choices and portions, and contributed to poor adherence to the suggested dietary changes. Although, the number of participants reporting these symptoms were not significantly different among the two groups. The physical activity i.e. minutes spent walking, had increased in both the groups, but the difference was not evident between groups.

Among the two arms, there is no significant difference in gestational weight gain, cesarean section, birth weight, or gestational age at birth as shown in Table 3. However, 93.4% of women in IL group gained weight within the IOM recommended as opposed to the UC group, where only 78.8% gained weight within the recommended target range. Furthermore, both groups reported two miscarriages each (LI = 6.6%, UC = 6%), and one stillbirth was reported in the LI group due to respiratory distress syndrome.

**Table 3:** Maternal pregnancy and neonatal outcomes

	Lifestyle Intervention group (*n* = 30)	Usual Care group (*n* = 33)	*P* value
Maternal pregnancy outcomes			
GDM (n)	10	19	0.05
Gestational weight gain (kg)	11.6 ± 5.11	13.2 ± 6.9	0.45
Exceeding IOM recommended weight gain n(%)	2 (6.6)	7 (21.2)	0.05
Fasting blood glucose (mmol/L)	5.04 ± 0.46	5.03 ± 0.63	0.93
Blood Glucose 1 hour	7.97 ± 1.76	8.85 ± 1.88	0.15
Blood Glucose 2 hour	7.15 ± 1.71	7.22 ± 1.72	0.89
Caesarean section, n (%)	11/28 (39)	13/23(47)	0.22
Neonatal outcomes			
Birth weight (g), mean (SD)	3193 ± 268	3185 ± 369	0.94
Birth weight > 4500 g, n (%)	0	0	–
Gestational Age at delivery (weeks)	38.4 ± 1.8	38.1 ± 3.4	0.69

*Notes*: Values are mean ± SDs or percentages. P-value derived from student t-test for continuous and chi square test for binomial data

*Abbreviations*: *GWG* Gestational weight gain, *IOM* Institute of Medicine

Incidence of GDM was 10/30 (33.3%) in the LI group and 19/33 (57.5%) in the UC group. The crude relative risk was 0.59 (95% CI, 0.32-1.04, *p* = 0.05). The results from multivariable logistic regression analysis (Table 4) indicated that a 12-week moderate intensity LI could decrease the odds of GDM by 63% (unadjusted) in high-risk pregnant women. However, the odds of developing GDM were reduced by 74% when adjusted for known confounders such as age, BMI, and family history of diabetes (OR = 0.26, 95%CI 0.07, 0.92, *p* = 0.037).

**Table 4:** Multivariate model analysis of unadjusted and adjusted for risk factors for GDM

Predictors	Model I unadjusted OR (95% CI)	*P* value	Model II adjusted OR (95% CI)	*P* value	Model III adjusted OR (95% CI)	*P* value
GDM	0.37 (0.13, 1.02)	0.05	0.29 (0.08, 0.96)	0.043	0.26 (0.07, 0.92)	0.04
Age	–		1.08 (0.96, 1.22)	0.177	1.10 (0.97, 1.26)	0.13
BMI	–		1.13 (1.00, 1.29)	0.048	1.13 (0.99, 1.29)	0.07
Family history T2D	–		–		2.12 (0.29, 15.2)	0.45
Area under ROC curve	0.621		0.745		0.752	
Pseudo R2	0.04		0.13		0.15	
p-value	0.052		0.012		0.01	

*Notes*: Model 1 was unadjusted; model II was adjusted for confounders (Age and BMI). Model III was adjusted for confounders in model II and family history of T2D. *P* < 0.05 assigned as significant, CI-Confidence Interval, OR-Odds Ratio

## Discussion

It was found that a 12-week moderate-intensity lifestyle intervention, delivered by a licensed dietitian through two individualized face-to-face sessions and two telephone sessions, could help reduce the risk of developing GDM among high risk pregnant women. This program focused on recommended weight gain, appropriate calorie intake, low-glycemic index meals, plant-based choices along with physical activity, and supported with behavior change cues.

Our results in high-risk pregnant women are encouraging, and similar results were reported in a recent meta-analysis of 40 studies, incorporating data from 30,871 high-risk pregnant women. According to this review, a Mediterranean diet and physical activity may be effective interventions for preventing diabetes. It was reported that diets resembling the Mediterranean diet, Dietary approaches to stop hypertension (DASH), and Alternate Healthy Eating Index diets were associated with 15-38% reductions in risk of GDM. The odds of developing GDM were also reduced 30 and 20% respectively by any physical activity during pregnancy or early pregnancy compared with no physical activity [23].

A Cochrane review of 23 randomized controlled trials on diet and exercise interventions for preventing GDM reported that the diet and exercise intervention group appeared to have a lower risk of gestational diabetes than the standard care group ((RR) 0.85, 95% (CI) 0.71 to 1.01) [24]. However, there is also evidence that suggests otherwise [25, 26]. It is believed that the lack of consensus over the results is due to the variability of the diet and exercise components tested, the duration of intervention, gestational week, and the selection criteria for participants. It is possible, however, that a selection of high-risk pregnant women could be one possible explanation for our results. Previous T2D prevention studies have shown a superior result in the high-risk group than in low or moderate group cohorts [9, 11]. The LI was initiated in the first trimester, which gave the participants a longer period of time to adhere to the recommended changes, which resulted in a positive outcome. Furthermore, this Lifestyle Intervention model has been demonstrated earlier to improve glycemic markers among diabetics and obese individuals in this population [17, 18].

The GWG was a reflection of total daily calorie intake by the end of pregnancy. When compared to UC, the LI group had 12% less GWG (LI = 3.2 vs 11.6 kg), and only 14.6% of participants exceeded the IOM recommendations of weight gain [19]. We need to recognize that since we recruited high-risk pregnant women, the women in the UC group also received general health advice on weight control during antenatal visits. This could contribute to narrowing the difference between the two groups.

The 12-week LI was initiated in the first trimester (6-12 gestation weeks) and showed a significant reduction in daily calorie intake (120 kcal/day, *p* = 0.004) compared to the UC group (46 kcal/day, *p* = 0.25). Weight gain within the IOM target range may explain the adherence to the recommended dietary changes throughout the pregnancy. However, we have used the validated self-reported 24-hour recall tool, which may underreport total calories as shown in earlier studies [27]. As part of the recommended diet, high levels of dietary fibre (vegetables, low-glycemic index fruits, beans, and whole grains) were recommended, as well as reduced refined carbohydrates (sugars, juices, white flour products), and restricted ultra-processed foods. These recommendations were very similar to the Mediterranean diet, which is a diet based upon plant-based foods, such as whole grains, vegetables, legumes, fruits, nuts, seeds, herbs, and spices. In addition, advised to consume fish, seafood, dairy products, poultry in moderation. Red meat and sweets are also to be consumed occasionally. As a result of nutritional intervention, the risk of GDM has reduced by 25% after adjusting for possible confounding factors [28].

While the intervention placed a greater emphasis on dietary intake than physical activity, it was found that adherence to physical activity recommendations (30%) was poor. Self-reported data were collected using a mobile phone pedometer. As a result of the LI group, the women reported an increase in daily physical activity of approximately 50 minutes. This indicated that small changes in calories and physical activity could reduce the risk of GDM in pregnant women. According to a meta-analysis of high-risk pregnant women, physical activity > 90 minutes per week appears to decrease the risk of gestational diabetes by 46% [23]. It is undisputed that moderate-intensity exercise with > 150 minutes per week can reduce the incidence of T2D and insulin resistance; physical activity below the recommended levels, or even light intensity, may also provide measurable health benefits [29].

There was no significant effect of the intervention on maternal or neonatal outcomes, including preterm delivery, birth weight, hypertension, mode of delivery, or macrosomia. Nonetheless, these outcomes are rare, as the small sample size could not adequately capture these outcomes due to their infrequency. A similar outcome was reported in the RADIEL trial on Finnish populations, where high-risk pregnant women experienced a 36% reduction in the risk of GDM; however, there was no effect on maternal and fetal outcomes [14].

Our study had its limitations; the recruitment was restricted to the government referral hospital in the region of Ajman, and the inclusion of high-risk pregnant women increased the internal validity. However, the generalizability is cautioned due to inclusion of a specific population group and inadequate power. Moreover, the data recorded for dietary intake, physical activity, and pre-gestational weight is self-reported hence attributed to recall bias. A regular GWG was not recorded, which prevented the study the variability of weight throughout pregnancy.

The nature of the intervention made it impossible to blind participants and providers, although blinding the data assessors reduced the risk of bias. A major strength of this study is its pragmatic design, primarily recruiting women who attended antenatal clinics and were referred to lifestyle clinics rather than requiring specialized resources. The short duration moderate intensity lifestyle intervention was reproducible in real-life situations. The ITT analysis of data enhanced the pragmatic nature of the study.

## Conclusion

To our knowledge, this is the first randomized controlled trial reported in this region that examines the effect of moderate-intensity lifestyle intervention on preventing GDM in high-risk pregnant women. We observed that the risk of developing GDM reduced by 41% among participants in the LI group as compared to UC group. Our findings emphasize that an individualized moderate-intensity lifestyle intervention in early pregnancy could reduce the risk of developing GDM among high-risk pregnant women in the UAE.

The success of this intervention steers the next question; whether lifestyle intervention should be integrated into the usual antenatal care for high-risk pregnant women. In a region where GDM and T2D are prevalent, this intervention could contribute to the well-being of pregnant women and reduce healthcare costs. However, further research is required to evaluate the effectiveness of this program as part of regular antenatal care and to evaluate its long-term effects on postpartum outcomes and its cost-effectiveness.

## Data Availability

The data used to support the findings of this study are restricted by the Ministry of Health and Prevention Research Ethics Committee–UAE, in order to protect patient privacy. Data are available from Amena Sadiya, Sadiya.amena@gmail.com for researchers who meet the criteria for access to confidential data.
